# Unsaturated or saturated dietary fat-mediated steatosis impairs hepatic regeneration following partial hepatectomy in mice

**DOI:** 10.1371/journal.pone.0284428

**Published:** 2023-05-11

**Authors:** S. M. Touhidul Islam, Arun P. Palanisamy, Gabriel R. Chedister, Michael G. Schmidt, David N. B. Lewin, Kenneth D. Chavin

**Affiliations:** 1 Department of Microbiology and Immunology, Medical University of South Carolina, Charleston, South Carolina, United States of America; 2 Department of Surgery, Case Western Reserve University School of Medicine, Cleveland, Ohio, United States of America; 3 Division of Transplant and Hepatobiliary Surgery, University Hospitals Cleveland Medical Center, Cleveland, OH, United States of America; 4 Division of Transplant Surgery, Department of Surgery, Medical University of South Carolina, Charleston, South Carolina, United States of America; 5 Department of Pathology and Laboratory Medicine, Medical University of South Carolina, Charleston, South Carolina, United States of America; 6 Department of Surgery, Temple University Hospital, Philadelphia, PA, United States of America; Kaohsiung Medical University, TAIWAN

## Abstract

**Background:**

Partial hepatectomy is a preferred treatment option for many patients with hepatocellular carcinoma however, pre-existing pathological abnormalities originating from hepatic steatosis can alter the decision to perform surgery or postoperative outcomes as a consequence of the impact steatosis has on liver regeneration.

**Aim:**

The aim of this study was to investigate the role of a saturated or unsaturated high fat diet-mediated steatosis on liver regeneration following partial hepatectomy.

**Methods:**

Mice were fed a low-fat control diet (CD, 13% fat), lard-based unsaturated (LD, 60% fat) or milk-based saturated high fat diet (MD, 60% fat) for 16 weeks at which time partial hepatectomy (approx. 70% resection) was performed. At days-2 and 7 post hepatectomy, one hour prior to euthanization, mice were injected with 5-bromo-2’-deoxyuridine in order to monitor hepatic regeneration. Serum was collected and assessed for levels of ALT and AST. Resected and regenerated liver tissue were examined for inflammation-indicative markers employing RT-PCR, Western blots, and histological methods.

**Results:**

Mice fed LD or MD exhibited higher NAFLD scores, increased expression of inflammatory cytokines, neutrophil infiltration, macrophage accumulation, increased apoptosis, and elevated levels of serum ALT and AST activities, a decrease in the number of BrdU-incorporated-hepatocytes in the regenerated livers compared to the mice fed CD. Mice fed MD showed significantly lower percent of BrdU-incorporated hepatocytes and a higher trend of inflammation compared to the mice fed LD.

**Conclusion:**

A diet rich in saturated or unsaturated fat results in NASH with decreased hepatic regeneration however unsaturated fat diet cause lower inflammation and higher regeneration than the saturated fat diet following partial hepatectomy in mice.

## Introduction

The liver has a unique ability to regenerate, even when mature, following a toxic insult or partial hepatectomy (PHx) [1, 2]. In spite of its substantial metabolic role, the liver is a quiescent organ in terms of hepatocyte proliferation. Although hepatocytes undergo a very low rate of proliferation under normal conditions, they can reach a dramatically high proliferation rate within the acinar architecture of the remnant liver following a surgical resection leading to total recovery of the organ within 2–6 weeks [3]. As a result of this ability to regenerate, surgical resection is often considered a viable treatment option for hepatocellular carcinoma (HCC) or end stage liver diseases including liver cirrhosis [4, 5].

Obesity is increasingly becoming a global health hazard with approximately 650 million adults warranting classification at being risk to the sequel associated with this condition [6]. In the United States, 34% of the adult population is clinically obese [7]. Emergence of the sedentary ‘corporate professional life’ and ‘fast food (containing high fat, carbohydrate, and calories) culture are considered to have contributed to this trend [8–10]. Although obesity has been correlated with heart disease, hypertension, colitis, diabetes type II, and nonalcoholic steatohepatitis (NASH), the role of diet and obesity in causing liver diseases demands further investigation. Hepatic steatosis, accumulation of fat in liver parenchyma, is very common in obesity [11]. Hepatic steatosis is usually considered innocuous due to its benign clinical consequences, but long-term steatosis can cause chronic inflammation, clinically represented as nonalcoholic steatohepatitis (NASH).

NASH serves as the bridge between steatosis and the onset of end stage liver diseases like cirrhosis and HCC [12, 13]. NASH is a key driver of non-viral HCC and NASH–HCC might be less responsive to immunotherapy [14] and surgical resection of part of the liver (PHx) is considered a viable treatment option for patients with end-stage liver diseases [4]. However post-resection regeneration capacity of NASH-HCC livers is a major concern. Therefore, it is important to have a clear understanding of the effect of any pre-existing pathological abnormality of liver, such as steatosis and NASH, on the hepatic regeneration following PHx.

Currently, no animal model perfectly mimics human hepatic steatosis. Commonly used animal models of hepatic steatosis involve genetic perturbations (e.g. leptin deficient ob/ob mice, leptin receptor deficient db/db mice, PTEN 10 null mice etc.) or feeding with MCD (diets deficient in methionine and choline). Each of these models manifest some metabolic features which are not consistent with the clinical paradigm of human hepatic steatosis [15]. However, a high fat diet, often referenced as “western diet”, mediated hepatic steatosis in mice is clinically more relevant [16]. In this study, we have assessed how hepatic steatosis induced by a diet, rich in either saturated or unsaturated fat, can lead to NASH and influence the hepatic regeneration post PHx.

## Methods

### Animals

Six-week-old male wild-type C57BL/6 mice were purchased from Jackson Laboratories. After two weeks of equilibration on normal chow, eight-week-old mice were placed either on an isocaloric control diet (TD.08810, 10% kcal from fat), a lard-based unsaturated high fat diet (TD.06414, 60% kcal from fat, 63% unsaturated) or a milk-based saturated high fat diet (TD.09766, 60% kcal from fat, 61% saturated), purchased from Harlan Laboratories (IN, USA). Sixteen animals were assigned for each diet feeding *ad libitum*. Mice were housed at 22°C with 12 h light/dark cycle, weighed every week and fed special diets for 16 weeks to induce steatosis. At 16-week time-point, they were subjected to PHx to assess the effect of steatosis on hepatic regeneration. This study was reviewed and approved by the Medical University of South Carolina’s Institutional Animal Care and Use Committee (IACUC) AR# 3003.

### Partial hepatectomy (PHx)

After 16 weeks of continuous feeding, mice were anesthetized with isoflurane and subjected to mid-ventral laparotomy with approximately 70% liver resection (left lateral and median lobes hereafter referred to as resected lobes) [17]. Animals from each diet group were sacrificed at 2 or 7 days post-hepatectomy. One hour prior to sacrifice, a single dose of 5-bromo-2’-deoxyuridine (BrdU, Sigma-Aldrich, MO, USA) was injected intraperitoneally at a dose of 50 mg/kg animal weight (0.2% solution in phosphate buffered saline). At the time of sacrifice, animals were re-anesthetized with isoflurane and total blood was harvested from the right ventricle of the heart, immediately followed by removal of the remaining liver lobes hereafter referred to as regenerated lobes. Both the resected and regenerated lobes of the liver were weighed and portions of them were processed for RNA and protein analyses. The remaining resected and regenerated lobes were fixed in 10% neutral buffered formalin (Starplex Scientific Inc., Ontario, Canada) for 24h at room temperature, with subsequent embedding in paraffin, sectioning, and histological/immunohistochemical staining.

### Serum analysis

Serum was prepared from whole blood using an established protocol [18] and assessed for the presence of alanine aminotransferase [ALT] (BioVision Inc., CA, USA) and aspartate aminotransferase [AST] (BioVision Inc., CA, USA).

### Liver histology and immunohistochemical staining

Formalin fixed paraffin embedded (FFPE) 5 μm sections were stained with Hematoxylin and Eosin (H&E). Slides were scored for the level of nonalcoholic fatty liver disease (NAFLD) by an experienced liver pathologist according to the semi-quantitative schema [19–21]. In brief, NAFLD activity was determined as a sum of steatosis, lobular inflammation, and hepatocellular ballooning scores. Levels of steatosis were determined from the amount (percent of area occupied) and size of empty spots in the liver sections left after the lipid-soluble ethanol wash during paraffin embedding and H&E staining. Lobular inflammation was determined depending on the level of cellular infiltration and hepatocellular ballooning was identified as enlarged hepatocytes (1.5–2 x diameter) with rarefied cytoplasm [19–21].

Fat droplets were visualized by oil red O (Sigma-Aldrich, MO, USA)-staining of sections form frozen tissues prepared in O.C.T. (Sigma-Aldrich, MO, USA) following standard protocol [22]. For picrosirius red staining of collagen, deparaffinized and rehydrated sections were incubated in a solution containing 0.1% Sirius red (Direct red 80, Sigma-Aldrich, MO, USA) and 1.3% saturated picric acid solution (Sigma-Aldrich, MO, USA). Sections were then washed in 0.5% acetic acid solution, dehydrated and cover slipped. All imaging was performed using ZEISS Axiovert 200M inverted microscope equipped with a digital camera.

Hepatocyte nuclear staining for BrdU was performed by using BrdU immunohistochemistry kit (abcam, MA, USA). BrdU-labeled positively stained hepatocytes were counted as percent of total cells in 10 high-powered microscope fields (HPF) per liver section.

For neutrophil counting, FFPE sections were stained by Leder stain (Napthol AS-D chloroacetate esterase, Sigma-Aldrich, MO, USA). The number of infiltrating neutrophils was counted per HPF for each section. For macrophage counting, F4/80 staining was performed by quenching of endogenous peroxidase activity by incubation of slides in 0.3% H_2_O_2_ for 30 min followed by incubation with primary antibody (rat anti-mouse F4/80, clone BM8, Biolegend, CA, USA) and immunostaining with Vectastain ABC kit (Vector Laboratories, CA, USA). Positively stained macrophages were counted as percent of total cells in 5 HPF per liver section [23].

### Quantitative real time RT-PCR

Total liver RNA was isolated using Trizol (ambion, CA, USA) according to the manufacturer’s instructions. The quality and concentration of RNA were confirmed by Nanodrop Spectrophotometer (Thermo Scientific, MA, USA) at 260/280 ratio. The concentrations of RNA between samples were normalized in RNase free water. For RT-PCR, 500 ng of RNA was first reverse transcribed and then amplified in a LightCycler 480 instrument (Roche, IN, USA) via a 1-step method using the TaqMan Fast Virus 1-step PCR master mix (Applied Biosystems, CA, USA) and TaqMan-FAM-MGB primers/probes assays (Applied Biosystems, CA, USA) using a 20 μL reaction volume according to the manufacturer’s protocol (primers/probes: HPRT: Mm00446968_m1, TNFα: Mm00443258_m1, IL1b: Mm00434228_m1, CCL2: Mm00441242_m1, TGFβ1: Mm00441724_m1, Col1a: Mm00801666_g1]) using thermocycling profile of reverse transcription at 50°C for 5 min, Reverse transcriptase inactivation/initial denaturation- 95°C for 20 sec, amplification-(95°C for 3 sec, 60°C for 30 sec) x40 cycles. Quantification of a given gene expression, expressed as relative mRNA level, was calculated after normalization to the housekeeping gene HPRT1 and calculated relative to the baseline control using the comparative ΔΔCt method.

### Immunoblotting

Total protein from the livers were used for Western blot analysis as described previously [24]. Briefly, liver tissues were homogenized in RIPA buffer containing 5% mammalian proteinase inhibitor (Sigma-Aldrich, MO, USA). Protein concentrations were determined by BCA assay (Pierce, IL, USA). 50 μg protein samples were run on 4–12% NuPage polyacrylamide gels (Life Technologies NY, USA) and transferred to nitrocellulose membranes. After blocking with TBS containing 0.05% Tween-20, and 5% milk for 30 minutes, blots were incubated overnight at 4°C with rabbit anti-PARP antibody (Cell signaling Technology, MA, USA) [25] or mouse anti-GAPDH antibody (Fitzgerald, MA, USA) diluted in TBS-T containing 5% milk at 1:1000 dilution. The blots were washed with TBS, incubated with goat anti-rabbit IgG or Horse anti-mouse IgG antibody (Cell signaling Technology, MA, USA) tagged with peroxidase at 1∶5000 dilution in TBS containing 5% milk (Pierce, IL, USA) for 1 hr at room temperature. After 3×5 min washing with TBST, blots were incubated with freshly prepared SuperSignal West Femto Maximum Sensitivity Substrate (Thermo Scientific, IL, USA) for 5 min and imaged using luminescent image analyzer (Image Quant LAS 4000, GE Healthcare Life Sciences, PA, USA). Densitometry analyses were performed using Image J software (National Institutes of Health, MD, USA).

### Statistical analyses

All values are expressed as mean ± standard error of the mean. Statistical significance was chosen *a priori* as α≤0.05. The non-parametric Mann-Whitney U test was used to analyze single, unpaired comparisons of normally distributed data sets. Statistical analyses were performed using GraphPad PRISM version 7.

## Results

### Mice fed LD or MD developed hepatic steatosis in both resected and regenerated livers

**Fig 1:**
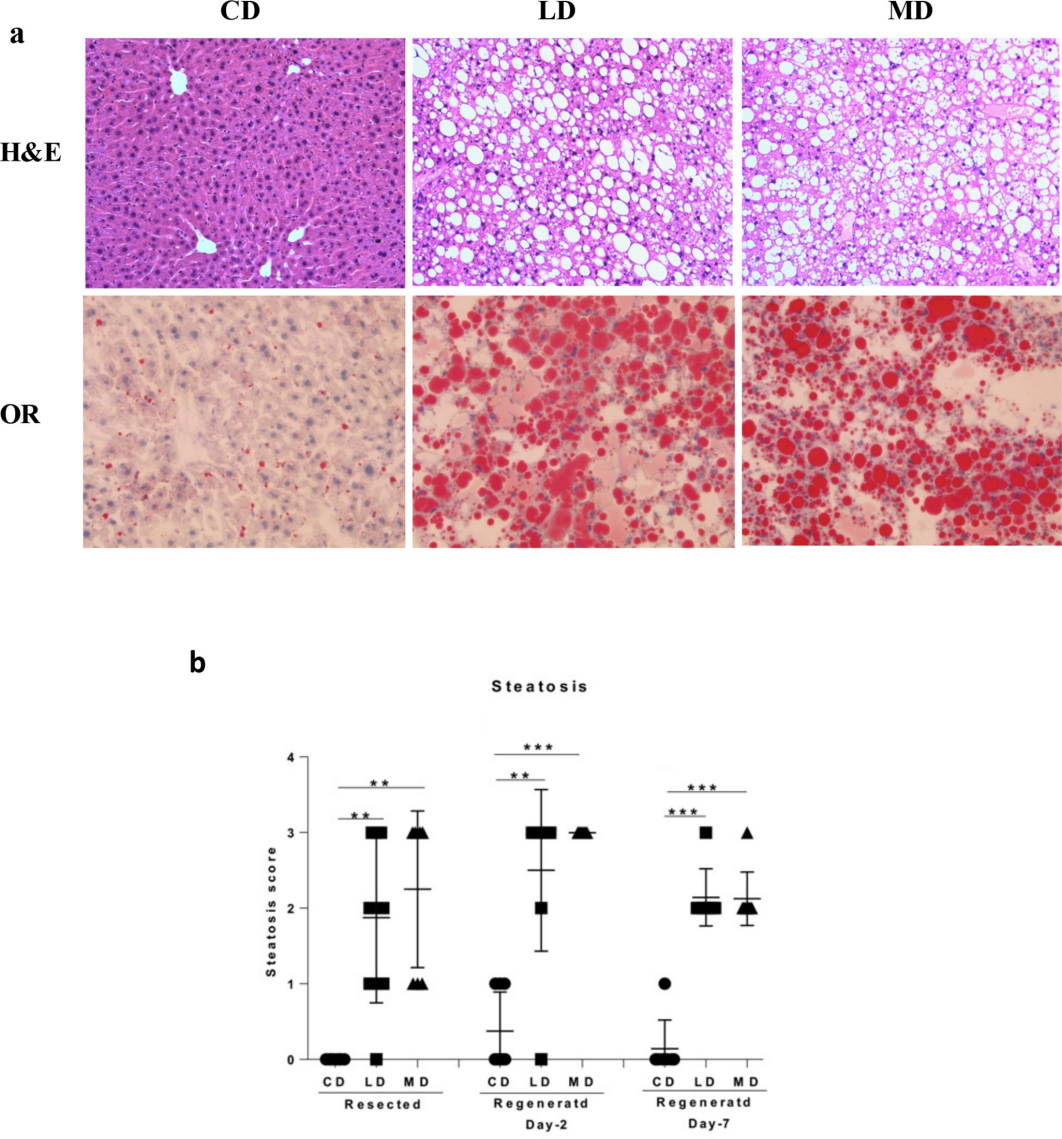
Consumption of unsaturated or saturated high fat diet led to hepatic steatosis in both resected and regenerated livers. Mice fed either a lard-based unsaturated or milk-based saturated fat rich diet for 16 weeks responded with fat accumulation in the liver as shown by the representative images of **(a)** H&E and **(b)** ORO stained regenerated day 2 liver sections and clinical score of steatosis. CD, LD, and MD denote control diet, lard-based unsaturated and milk-based saturated high fat diet respectively. *p<0.05, **p<0.01, ***p<0.001 (Man-Whitney test); data are expressed as mean±SEM; n = 6–8.

Subsequent to the feeding of mice for 16 weeks, partial hepatectomy was performed resulting in surgical resection of 70% of the livers. Animals within the control and experimental groups were allowed to recover from the partial hepatectomy for either 2 or 7 days. H&E and ORO stained images of the liver sections clearly demonstrated fat deposition in resected (S1a Fig in S1 File), regenerated day-2 (Fig 1A), and regenerated day-7(S1B Fig in S1 File) livers of mice fed unsaturated (LD) or saturated (MD) high fat diet compared to those of mice fed control diet (CD). Fat accumulation in the livers of mice fed high fat diet was reflected in their clinical score of steatosis. Both resected and regenerated livers from the mice fed LD or MD displayed higher clinical scores of steatosis compared to the mice fed CD diet (Fig 1B).

### Long term steatosis led to the development of NASH and fibrosis in both resected and regenerated livers

**Fig 2:**
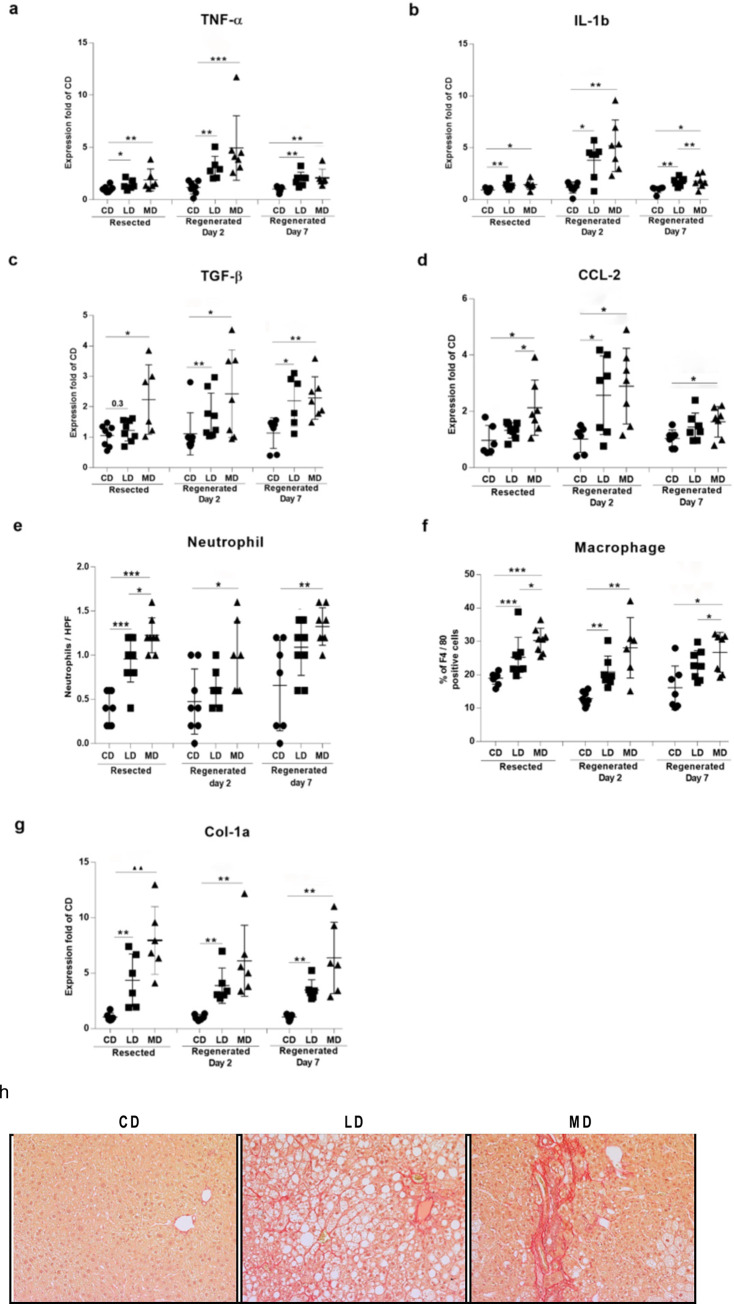
Unsaturated or saturated high fat diet-mediated hepatic steatosis led to the development of NASH and hepatic fibrosis in both resected and regenerated livers. Hepatic steatosis in mice fed either a lard-based unsaturated or milk-based saturated high fat diet for 16 weeks resulted in the development of NASH as evidenced by increases to the expression of pro-inflammatory cytokines: **(a)** TNF-α, **(b)** IL-1β, **(c)** TGF-β; **(d)** chemokine CCL-2; **(e)** increased infiltration of neutrophil and **(f)** accumulation of macrophage. Hepatic steatosis in mice fed either a lard-based unsaturated or milk-based saturated high fat diet for 16 weeks also resulted is hepatic fibrosis as evidenced by the increased level of **(g)** expression of Col1 and **(h)** representative images of picrosirius red stained regenerated day-2 liver sections. CD, LD, and MD denote control diet, lard-based unsaturated and milk-based saturated high fat diet respectively. *p<0.05, **p<0.01, ***p<0.001 (Man-Whitney test); data are expressed as mean±SEM; n = 6–8.

Progression of high fat diet-mediated steatosis to NASH was examined by determining the levels of different inflammation-indicative markers. mRNA analysis of different pro-inflammatory cytokines and chemokines (TNF-α, IL-1β, TGF-β, and CCL-2) showed higher levels of expression in both of the resected and regenerated livers of mice fed LD or MD compared to the mice fed CD (Fig 2A–2D). Hepatic infiltration of different myeloid immune cells exhibited higher infiltration of neutrophils (Fig 2E) and F4/80^+^ macrophages (Fig 2F) in both of the resected and regenerated livers of mice fed LD or MD as compared to the mice fed CD. The level of fibrosis, an important indicator of chronic inflammation in fatty liver, [21] was also determined by investigating collagen accumulation. Higher level of expression of collagen-1a was exhibited in the resected and regenerated livers of mice fed LD or MD (Fig 2G). Accumulation of collagen was also observed in the picrosirius red-stained sections from resected (S2A Fig in S1 File), regenerated day 2 (Fig 2H), and regenerated day 7 (S2B Fig in S1 File) livers of mice fed LD or MD. All of these indicators clearly demonstrated the development of NASH in mice fed LD or MD. Noticeably, livers in mice fed MD trended to exhibit higher levels of expression of inflammatory cytokines (Fig 2A–2D), infiltration of neutrophils and macrophages (Fig 2E and 2F), and an accumulation of collagen (Fig 2G and 2H) compared to those exhibited by in the livers of mice fed LD. These data suggest that animals fed MD will experience a greater risk of developing NASH-morbidity in both resected and regenerated livers compared to animals fed LD diet.

### Long term steatosis caused damage in both resected and regenerated livers

**Fig 3:**
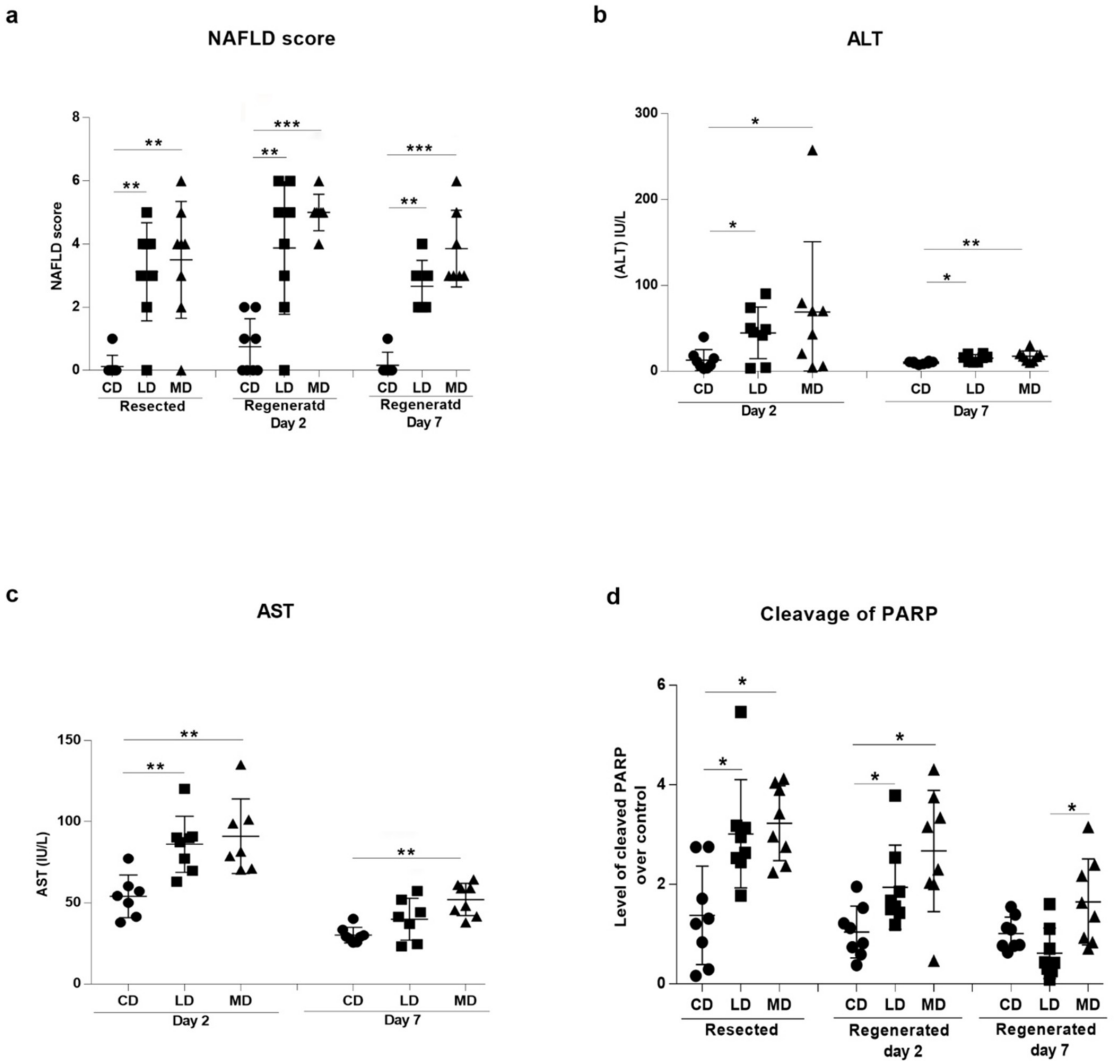
High fat diet mediated-hepatic steatosis resulted in increased liver damage in both resected and regenerated livers. Dietary fat mediated hepatic steatosis resulted in higher **(a)** NAFLD scores and elevated level of serum **(b)** ALT and **(c)** AST. **(d)** Densitometry analyses of immunoblots exhibited increased level of cleavage of poly ADP-ribose polymerase (PARP) indicating increased level of apoptosis in steatotic livers. CD, LD, and MD denote control diet, lard-based unsaturated and milk-based saturated high fat diet respectively. *p<0.05, **p<0.01, ***p<0.001 (Man-Whitney test); data are expressed as mean±SEM; n = 6–8.

H&E stained liver sections were graded for clinical scores of NAFLD as an indication of the severity of inflammation in fatty livers. Both of the resected and regenerated liver sections from mice fed LD or MD displayed higher NAFLD scores compared to the mice fed CD (Fig 3A). Liver sections from the mice fed MD trended to have higher NAFLD scores compared to the mice fed LD (Fig 3A). Sera from mice fed LD or MD showed increased levels of ALT (Fig 3B) and AST (Fig 3C) both on day-2 or day-7 compared to the mice fed CD. This indicated higher levels of liver damage in the mice fed unsaturated or saturated high fat diets. When compared between the mice fed LD and MD, sera from the mice fed MD showed a higher trend of ALT and AST levels both on day 2 and day 7 compared to the mice fed LD (Fig 3B and 3C).

Apoptosis resulting from the NASH-mediated hepatic tissue injury was determined by comparing the level of cleaved poly ADP-ribose polymerase (PARP). Both resected and regenerated tissues on day 2 or day 7 from the mice fed MD showed an increased level of cleaved PARP compared to the mice fed CD (Fig 3D, Western blot images in S3A-S3F Fig in S1 File). Mice fed LD showed an increased level of cleaved PARP in the tissues from the resected and regenerated day 2 livers compared to the mice fed CD (Fig 3D). However, mice fed MD displayed a higher trend of PARP cleavage compared to the mice fed LD in both resected and regenerated livers on day 2 and day 7 (Fig 3D).

### Steatotic livers demonstrated impaired regeneration following partial hepatectomy

**Fig 4:**
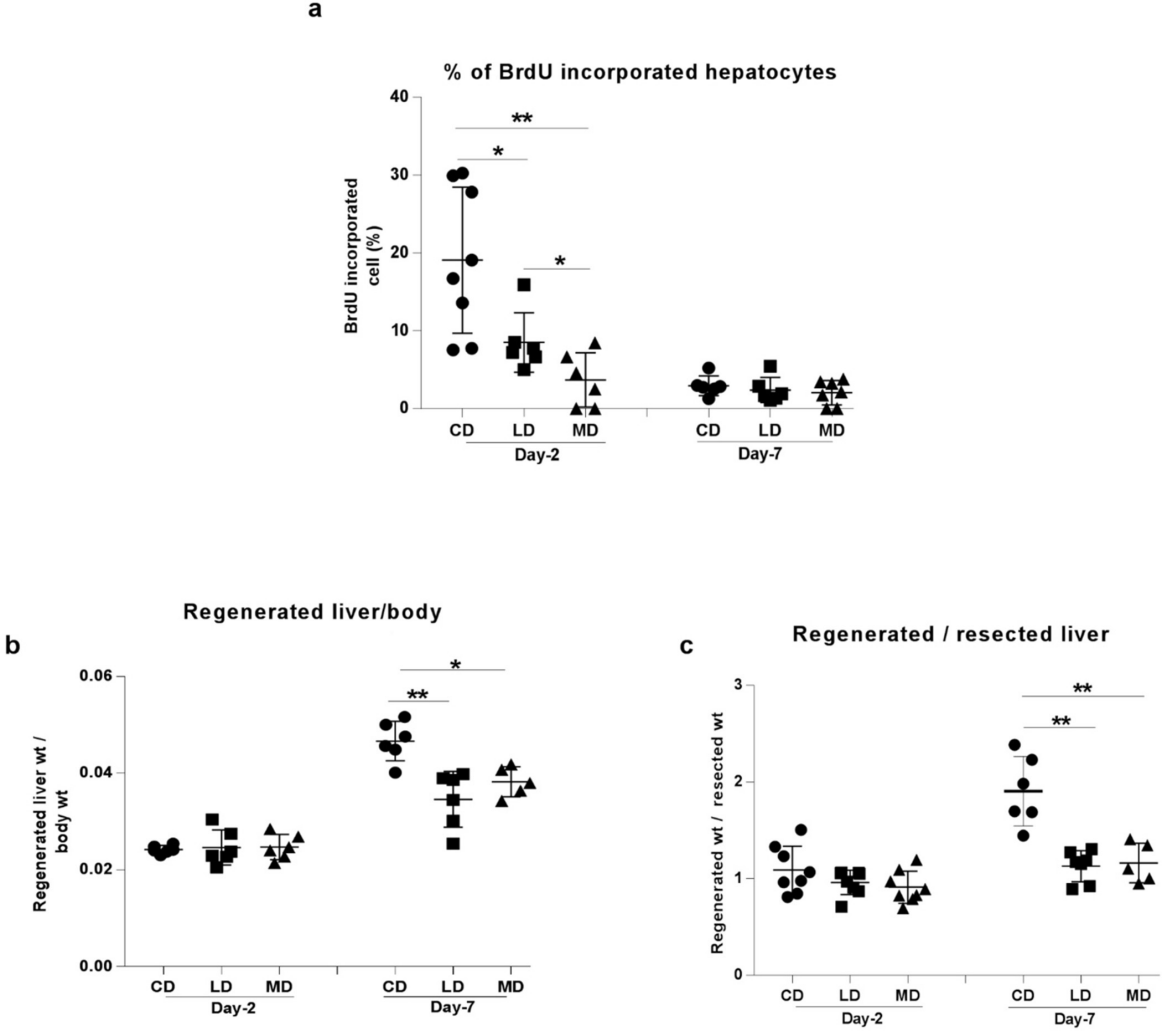
Hepatic regeneration following partial hepatectomy was impaired in NASH affected liver. NASH induced through the long term consumption of high fat diets resulted in impaired regeneration of partially resected livers as evidenced by **(a)** reduced rates of BrdU incorporation in hepatocytes, decreased **(b)** regenerated liver/body ratio and **(c)** regenerated liver/resected liver ratio. CD, LD, and MD denote control diet, lard-based unsaturated and milk-based saturated high fat diet respectively. *p<0.05, **p<0.01 (Mann-Whitney U test); data are expressed as mean±SEM; n = 6–8.

Percentage of BrdU-incorporated hepatocytes was determined in regenerated liver sections on day 2 and day 7 in order to assess the effect of NASH on hepatic regeneration following partial hepatectomy. The percentage of BrdU-incorporated hepatocytes was 2.2 and 5.2 fold lower in the regenerated liver sections of mice fed LD or MD respectively compared to that of the regenerated liver sections of mice fed CD on day 2 (Fig 4A). These data demonstrate the impaired hepatic regeneration in mice fed fat-rich diets of either types compared to the mice fed control diet. Of note, percent of BrdU-incorporated hepatocytes was 2.3-fold lower in mice fed MD compared to the mice fed LD. This observation was consistent with the higher trending indicators of the development of NASH (Fig 2), hepatic damage (Fig 3A–3C) and apoptosis (Fig 3D and 3E) in the livers of the mice fed MD when compared to the livers of mice fed LD. Because hepatic regeneration following PHx reached at saturation by day 7 [26], no significant difference in BrdU incorporation was observed at day 7. However, the percentage of incorporation of BrdU trended to be lower in the livers of mice fed LD or MD compared to that incorporated into the livers of mice fed CD.

Regenerated liver output was measured as the ratio of regenerated liver-to-total body weight (Fig 4B) or regenerated liver-to-resected liver weight (Fig 4C). In either case, ratios were lower in mice fed LD or MD compared to those of mice fed CD on day 7. Regenerated livers from the mice fed LD or MD showed a 1.3 and 1.2-fold reduction of regenerated liver-to-body weight ratio respectively compared to the mice fed CD (Fig 4B). Regenerated livers from the mice fed LD or MD were found to have 1.7 and 1.6 fold reduction in their regenerated liver-to-resected liver weight ratio respectively compared to the mice fed control diet (Fig 4C). These data again corroborated the impaired regeneration of NASH-affected livers following PHx.

## Discussion

Our current data primarily demonstrate that long-standing hepatic steatosis in mice fed a high fat diet leads to NASH and impairs hepatic regeneration following PHx. NASH is expected to surpass hepatitis C as the dominant cause of liver failure and is also becoming the most common cause of HCC in the US [27–29]. Although liver transplantation has opened a new avenue for the treatment of HCC, the benefit of this treatment is limited mainly by shortage of available organs and also long-term complications such as immunosuppression-related infections, risk of tumor progression etc. Therefore, PHx is still considered as the most viable treatment for HCC [30]. Because of the co-existence of NASH and HCC in a large number of patients [31, 32], it is important to investigate how the presence of NASH would affect hepatic regeneration in these patients following PHx. Findings of this study may influence the decision to perform and/or post-operative management of NASH patients being considered for this surgery. Absence of an appropriate animal model that effectively mimics the genesis of human NASH represents a barrier in being able to appreciate the consequences that NASH has on hepatic regeneration following surgical resection. However, amongst the currently available genetic and dietary models, dietary fat induced NASH is clinically more relevant. Previously, we have shown how employing a model requiring significant exposure to a fat-rich diet was sufficient to induce and then mimic the complex behavior associated with NASH that closely mimics what occurs in humans using LD and MD diets [33].

Comparison between the impacts of unsaturated and saturated dietary fat induced NASH on hepatic regeneration was an additional aspect of this study. The data collected provided evidence for a trend towards higher levels of expression of pro-inflammatory cytokines, increases in NAFLD score, collagen accumulation, serum ALT/AST levels and apoptosis in the livers of mice fed saturated high fat diet (MD) compared to the mice fed unsaturated high fat diet (LD) indicating an exacerbating role of a diet high in saturated fats in causing NASH. An exacerbation of impairment of hepatic regeneration as evidenced by of BrdU-incorporated hepatocytes on day 2 following PHx in the fatty livers of mice fed MD was consistent with a higher trend of hepatic inflammation and hepatocyte damage compared to the mice fed LD. Such a higher trend of hepatic inflammation can be attributed to aggravated oxidative stress, lipotoxicity, and endoplasmic reticulum stress mediated by the saturated fat [34, 35]. Chronic endotoxemia might also be plausible in explaining higher inflammation in mice fed MD. It is yet to be clarified whether chronic endotoxemia occurs due to the shifting of gut microbiota to a higher endotoxemic profile or results from an increased intestinal permeability in mice fed high fat diet.

We have observed that germ free mice or broad-spectrum antibiotic-treated mice fed LD or MD are partially protected from NASH (unpublished data). Therefore, it is important to investigate if diminishing the gut microbial population in mice fed high fat diet through the treatment with broad spectrum antibiotics can improve the impairment of hepatic regeneration following surgical resection. Changes in factors that are involved in the poor regeneration of livers with NASH including dysfunction of the ER stress response [36], inhibition of β-oxidation [37] different localization of autophagosomes in the fatty liver [38] would provide more mechanistic insight. It would also be interesting to study if the treatment of mice with a NASH-reducing drug can improve the impairment of hepatic regeneration post PHx to make surgical resection a more viable treatment option for end stage liver diseases.

## Conclusion

A diet rich in saturated or unsaturated fat results in NASH with decreased hepatic regeneration following partial hepatectomy in mice with saturated fat diet having a more pronounced effect on early regeneration.

## Supporting information

S1 File (DOCX)

S1 Raw images (PDF)
