# Supplementary material for: Unsaturated or saturated dietary fat-mediated steatosis impairs hepatic regeneration following partial hepatectomy in mice
Source: PLoS One. 2023 May 11;18(5):e0284428. doi: 10.1371/journal.pone.0284428 (PMC10174548; doi:10.1371/journal.pone.0284428)
Supplement: S1 File — (DOCX) [file pone.0284428.s001.docx]

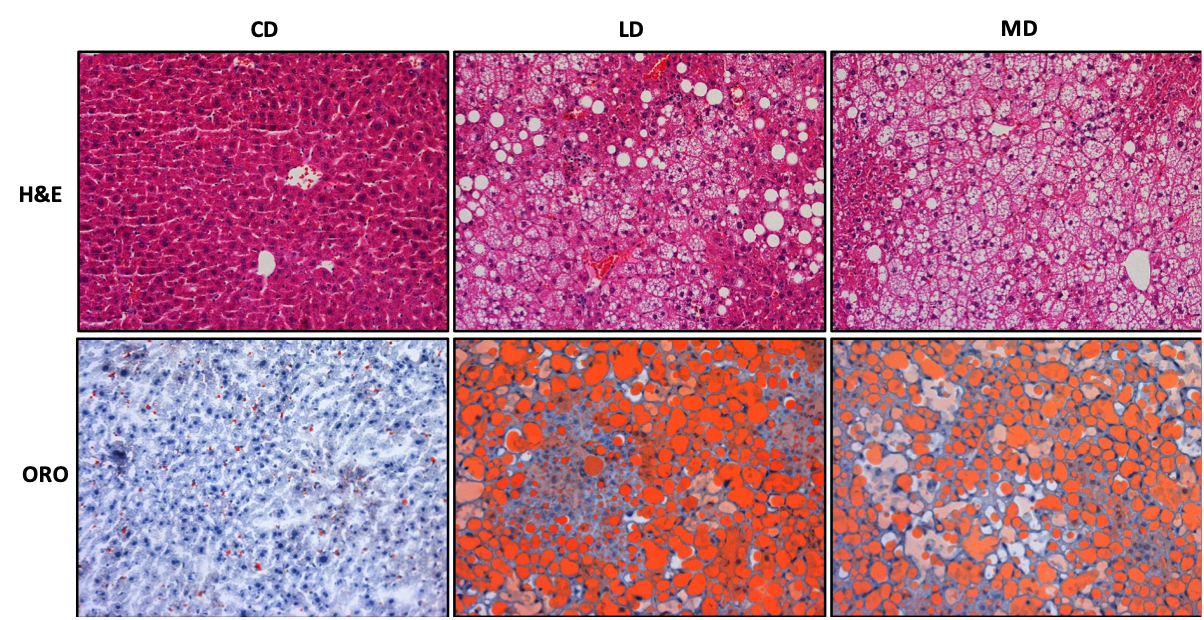
**a**


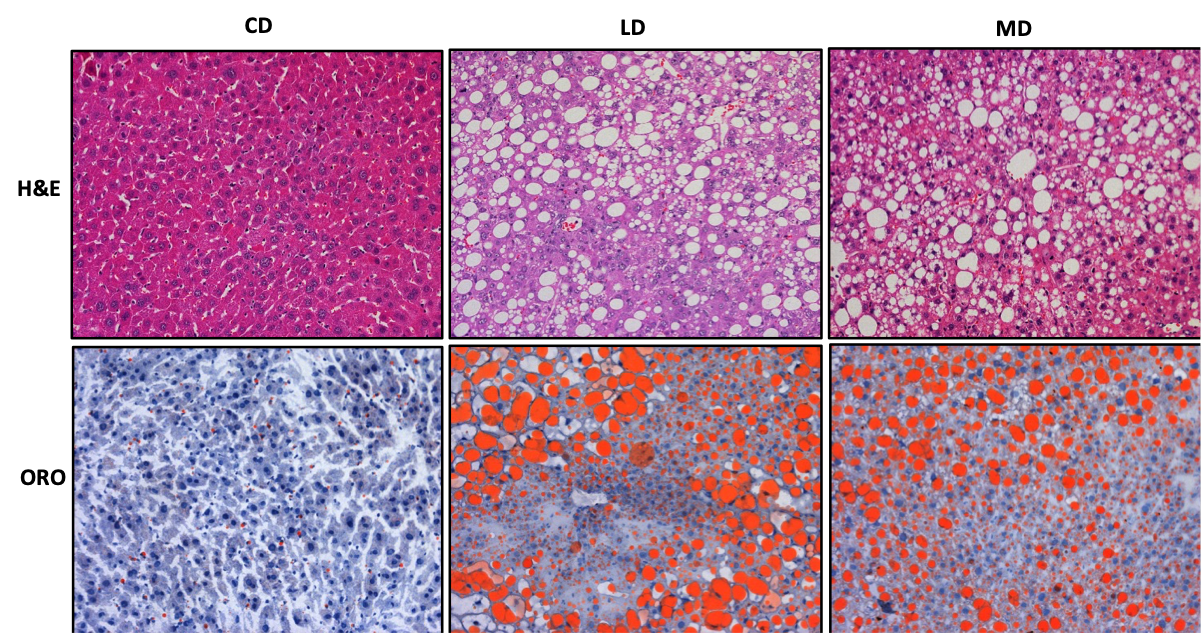
**b**

**Supplementary figure 1. Mice fed unsaturated or saturated high fat diet developed steatosis in both resected and regenerated livers.** Feeding of mice with either unsaturated or saturated high fat diet resulted in the development of steatosis in both resected and regenerated livers as shown by the representative images of H&E and ORO stained **(a)** resected and **(b)** regenerated day 7 liver sections. CD, LD and MD denote control diet, lard-based unsaturated and milk-based saturated high fat diet respectively.

**a**


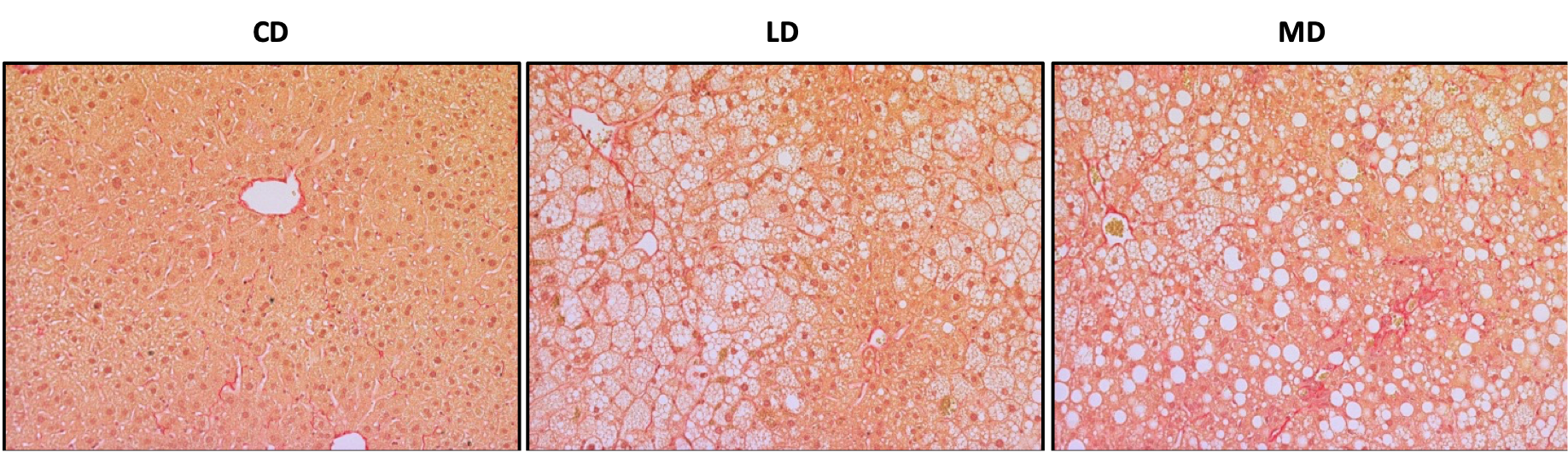


**b**


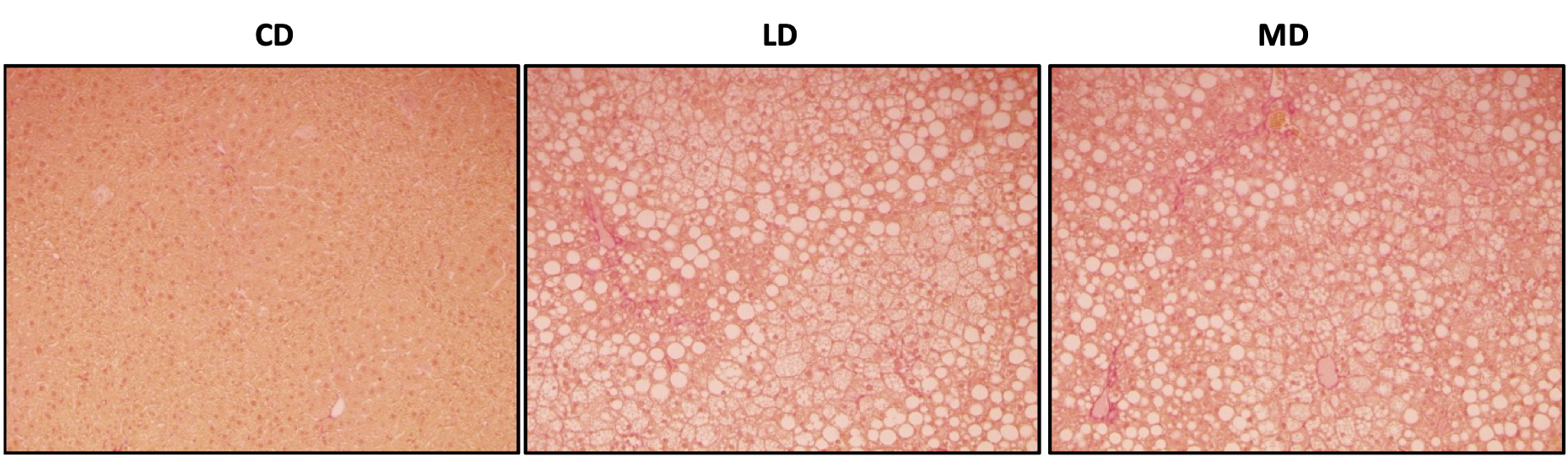


**Supplementary figure 2. Mice fed unsaturated or saturated high fat diet exhibited increased collagen accumulation in both resected and regenerated livers.** Feeding of mice with either unsaturated or saturated high fat diet resulted in higher level of accumulation of collagen in both resected and regenerated livers as shown by the representative images of picrosirius red stained **(a)** resected and **(b)** regenerated day 7 liver sections. CD, LD and MD denote control diet, lard-based unsaturated and milk-based saturated high fat diet respectively.

**a**


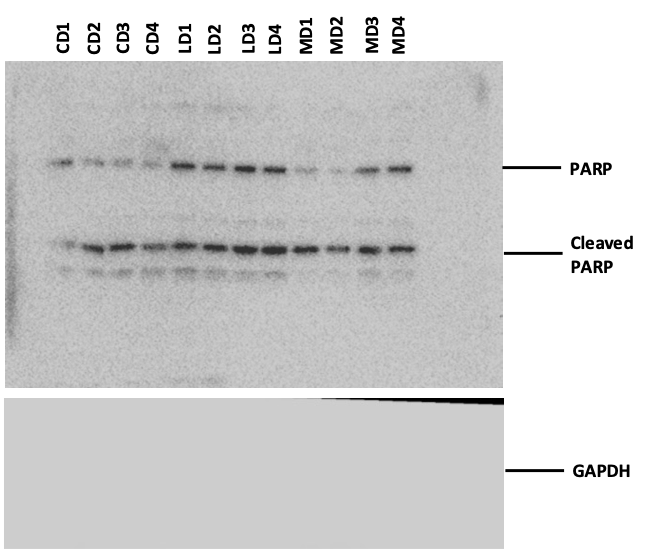
**b**


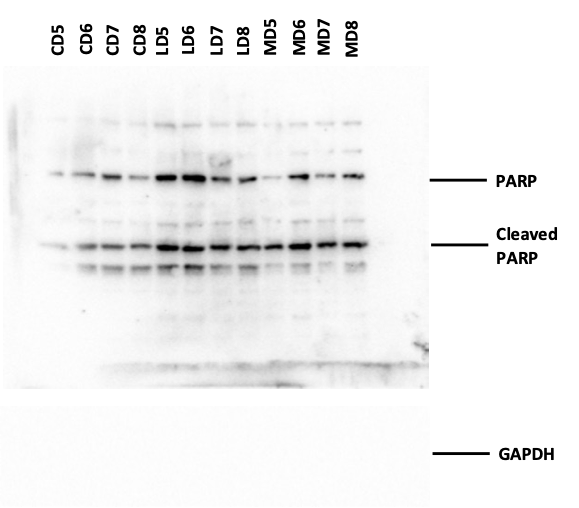


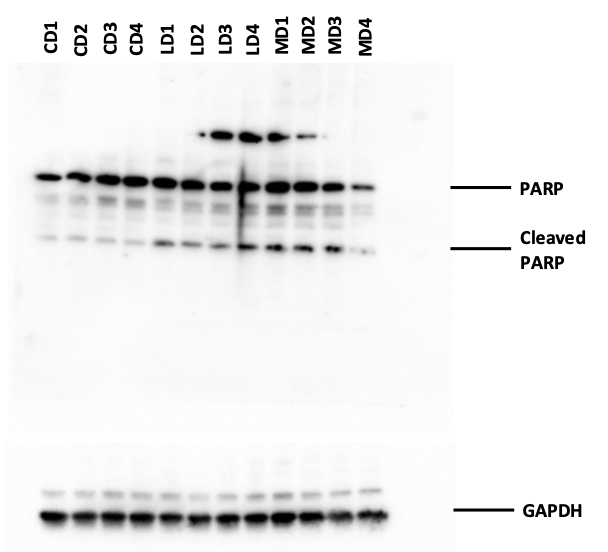

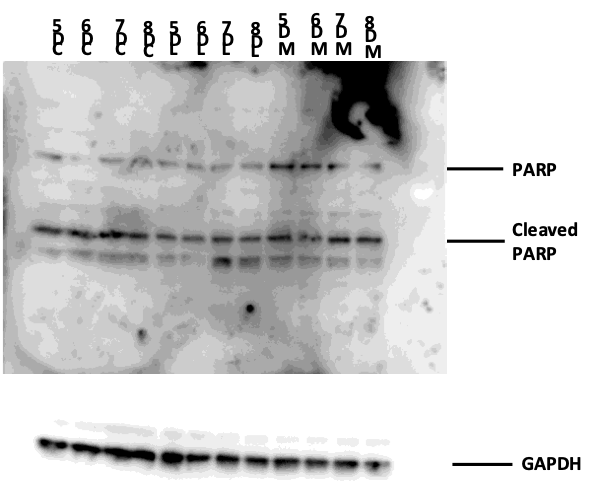
**c d**


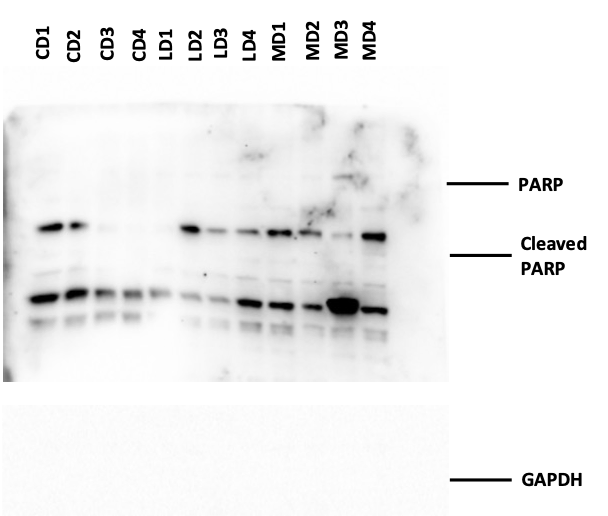
**e**


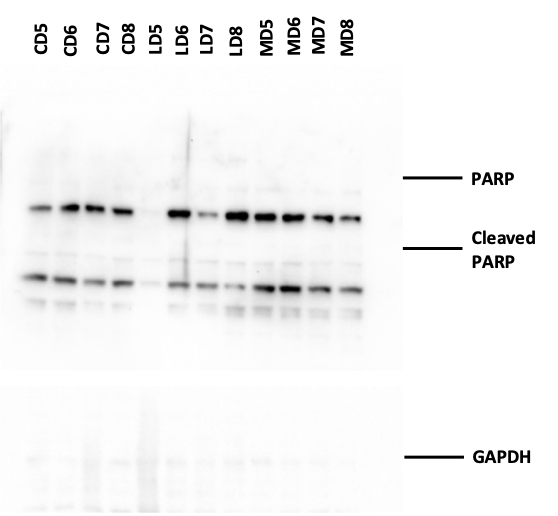
**f**

**Supplementary figure 3. Western blot images demonstrated higher level of cleavage of poly ADP-ribose polymerase (PARP) in the resected and regenerated livers of mice fed unsaturated or saturated high fat diet.** Western blot analyses resulted in higher level of cleavage of PARP in the **(a-b)** resected, **(c-d)** regenerated day 2 and **(e-f)** regenerated day 7 livers of mice fed unsaturated or saturated high fat diet compared to the mice fed control diet. CD, LD and MD denote control diet, lard-based unsaturated and milk-based saturated high fat diet respectively; n=8.
