# Supplementary figures and images for: Unsaturated or saturated dietary fat-mediated steatosis impairs hepatic regeneration following partial hepatectomy in mice
Source: PLoS One. 2023 May 11;18(5):e0284428. doi: 10.1371/journal.pone.0284428 (PMC10174548; doi:10.1371/journal.pone.0284428)

**a**

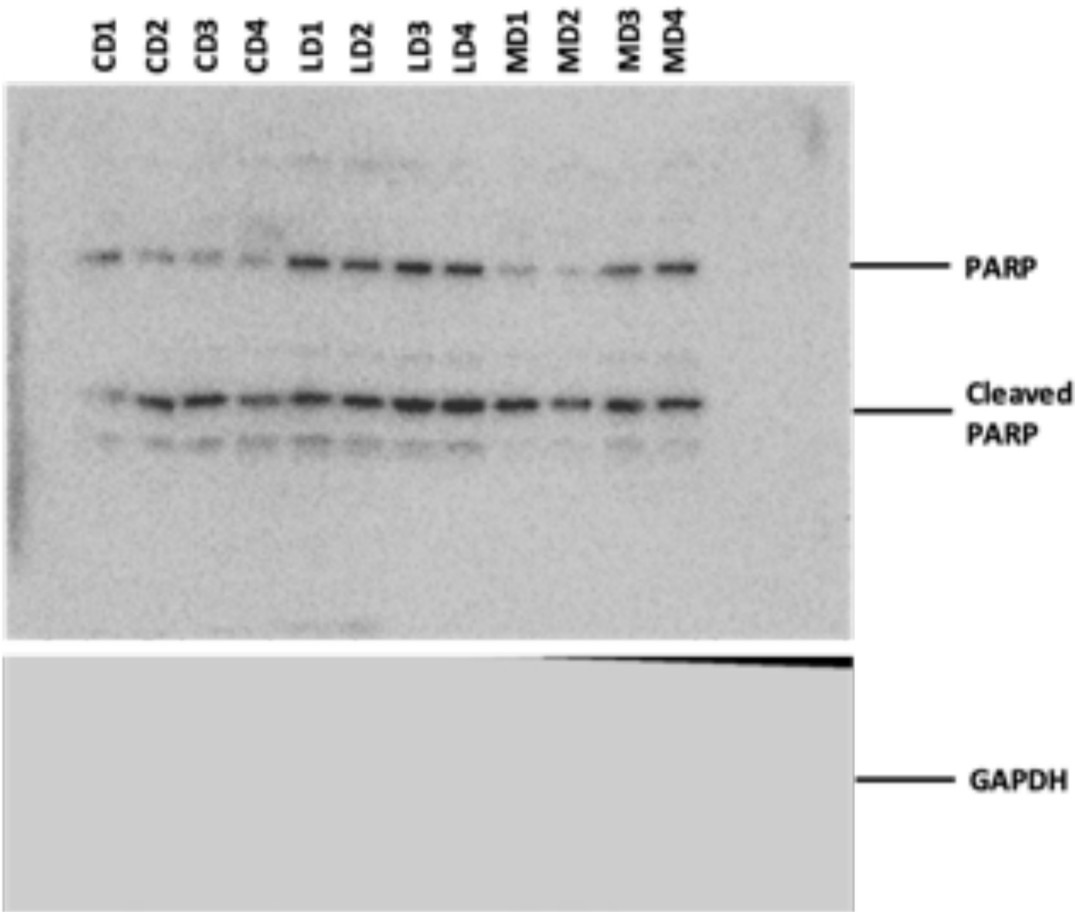

**b**

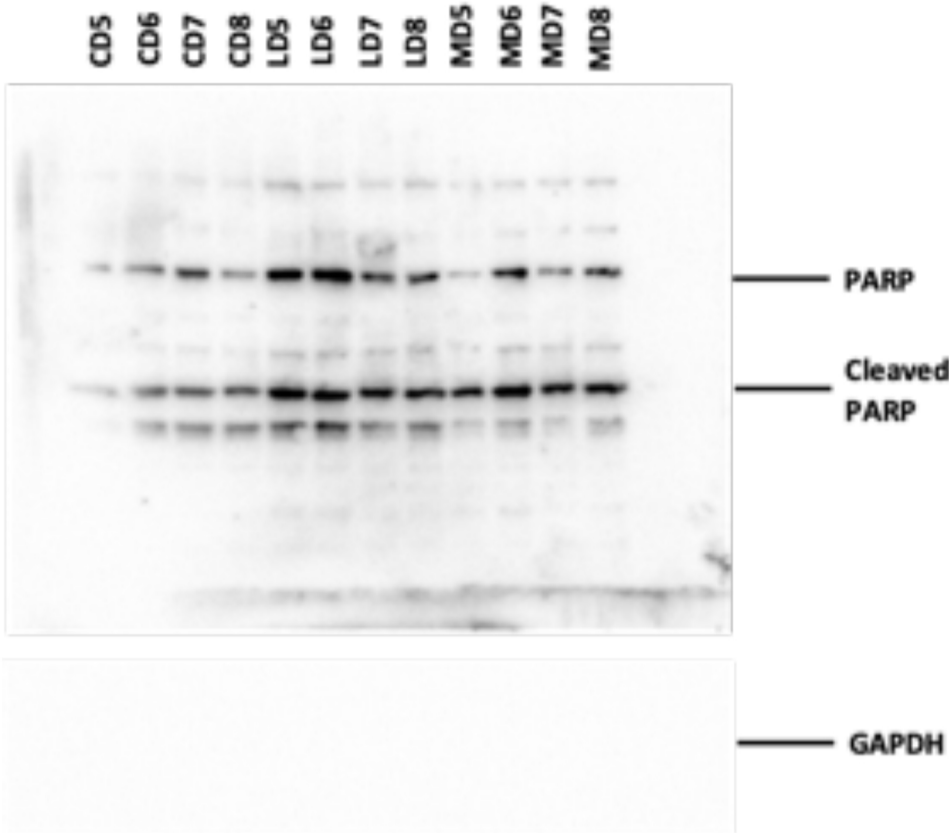

**c**

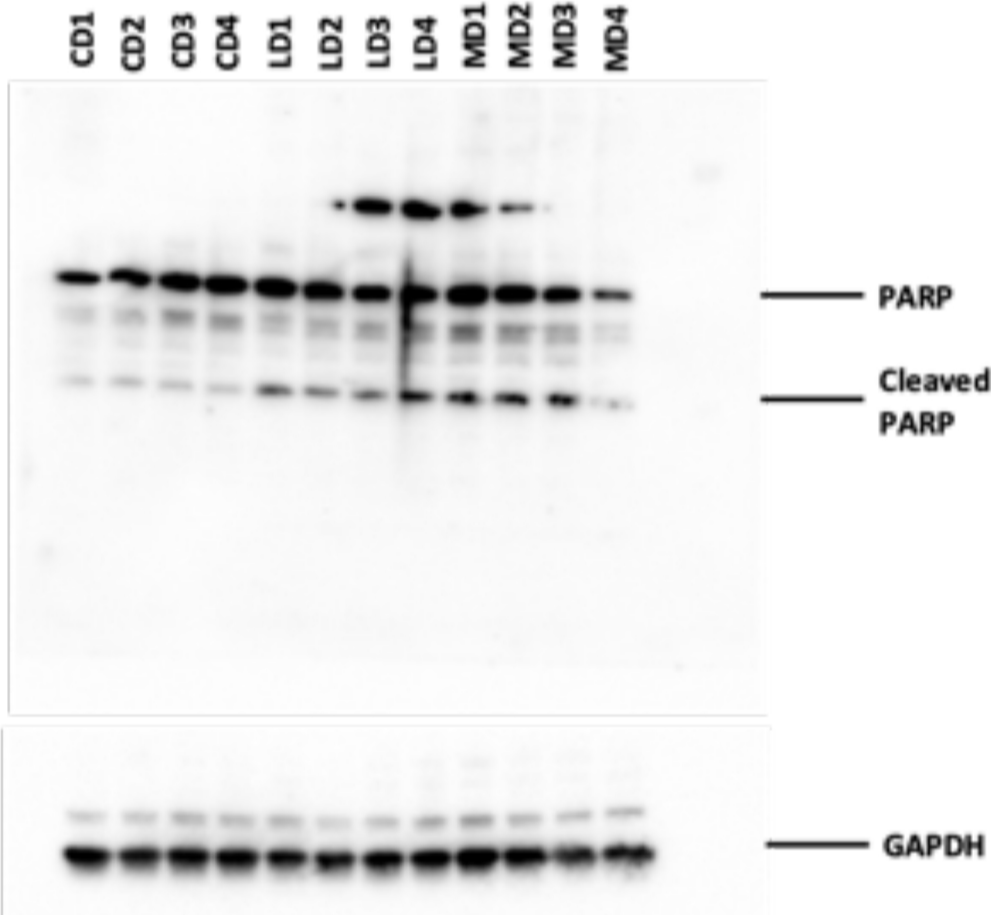

**d**

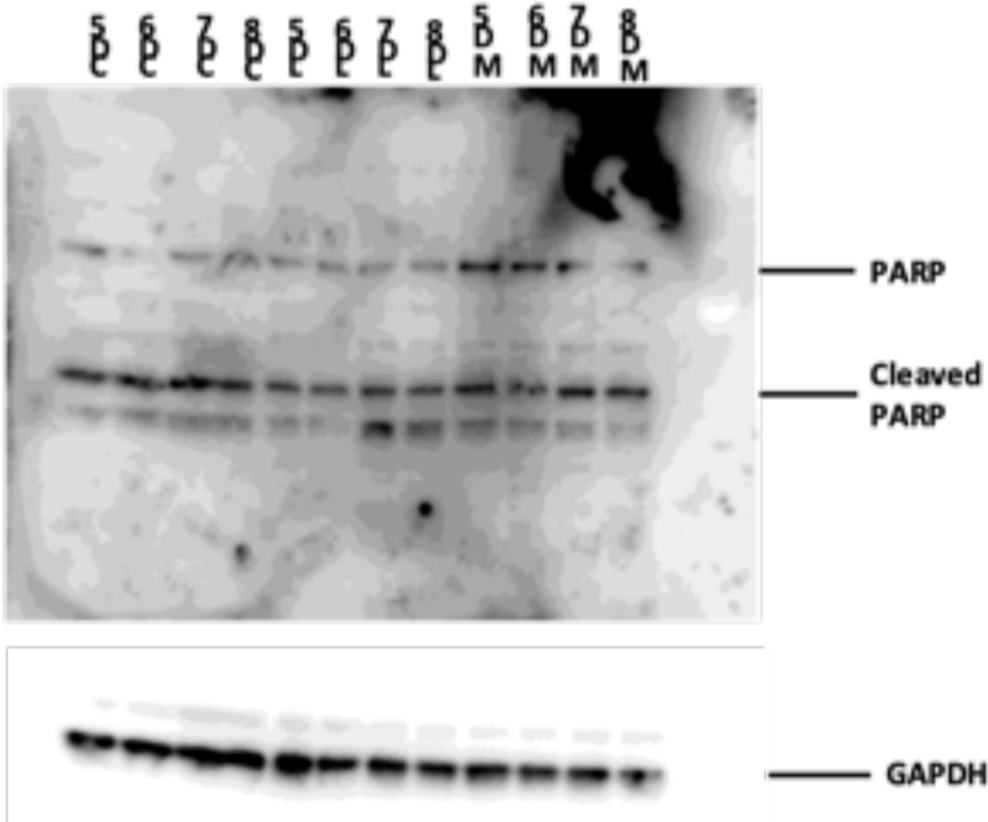

**e**

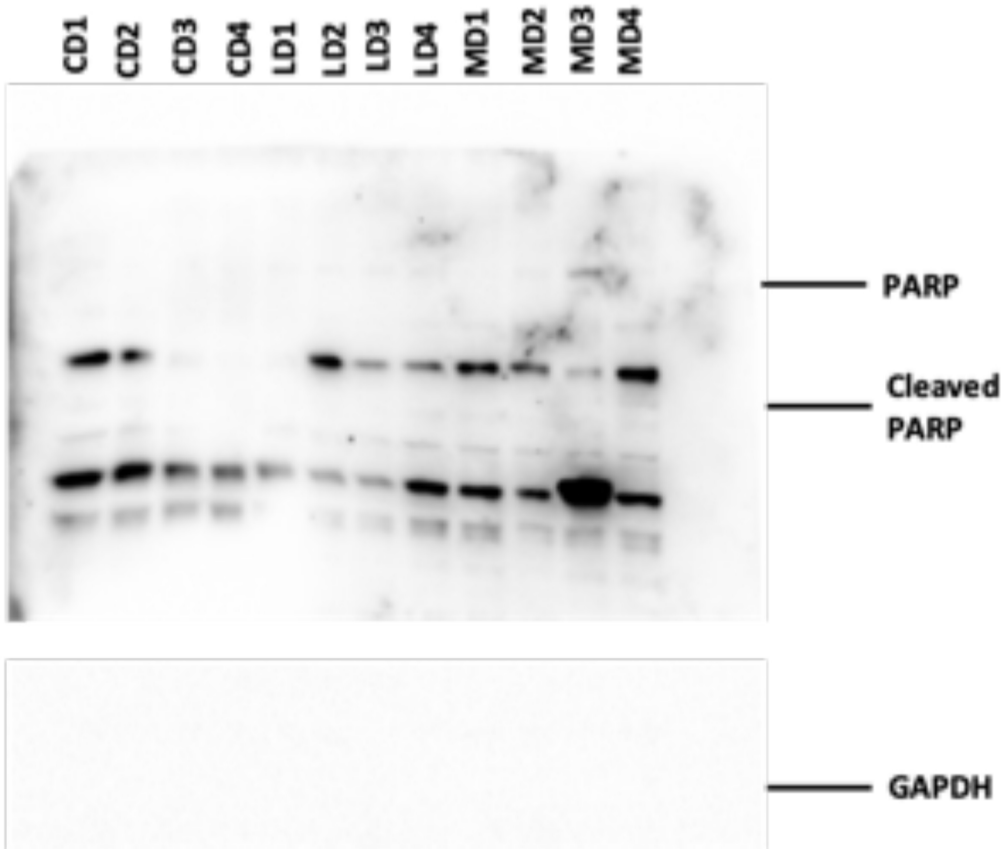

**f**

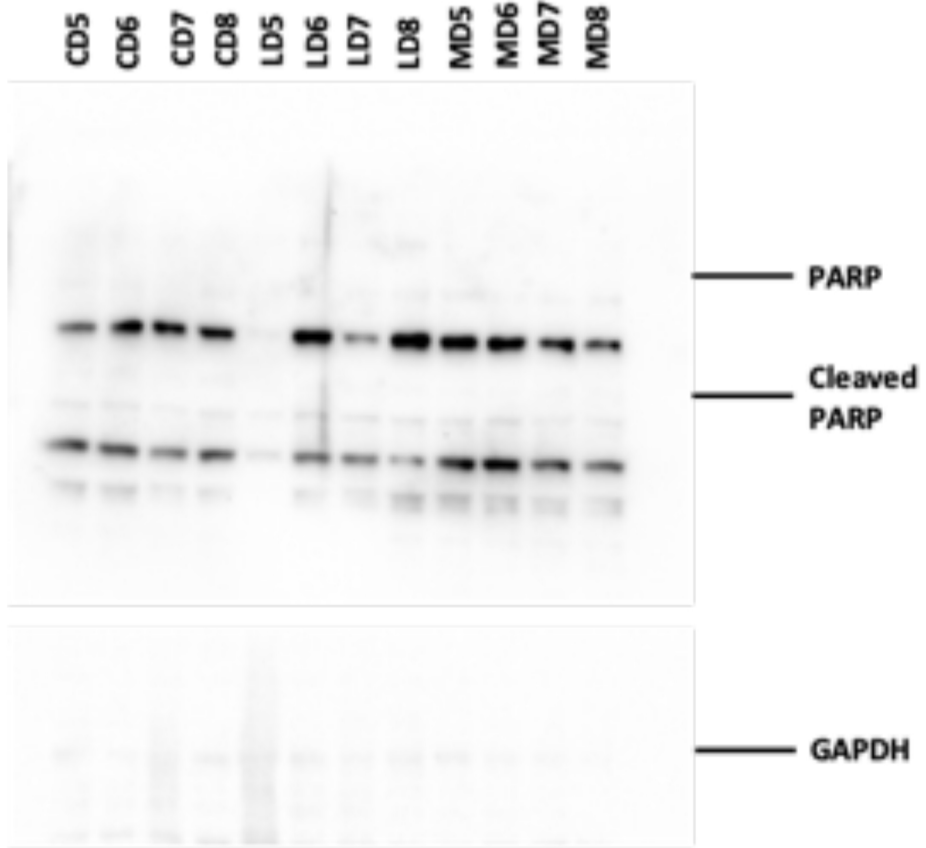

Supplement: S1 Raw images — (PDF) [file pone.0284428.s002.pdf]
